# Family caregivers as essential partners in care: examining the impacts of restrictive acute care visiting policies during the COVID-19 pandemic in Canada

**DOI:** 10.1186/s12913-023-09248-3

**Published:** 2023-03-31

**Authors:** Stephanie Montesanti, Gail MacKean, Kayla M. Fitzpatrick, Carol Fancott

**Affiliations:** 1 School of Public Health, University of Alberta, Edmonton, AB Canada; 2 Centre for Healthy Communities, School of Public Health, University of Alberta, Edmonton, AB Canada; 3 Imagine Citizens Network, Calgary, AB Canada; 4 Department of Community Health Sciences, University of Calgary, Calgary, AB Canada; 5 Healthcare Excellence Canada, Ottawa, ON Canada

**Keywords:** Family caregivers, Restrictive visiting policies, Family presence, Acute care, COVID-19, Canada

## Abstract

**Introduction:**

During the pandemic many Canadian hospitals made significant changes to their ‘open family presence’ and ‘visitor policies’ to reduce the spread of COVID-19 by instituting restrictive or ‘zero visiting’ policies in healthcare facilities. These policies have the potential to create great hardship, anxiety and stress for patients, families, caregivers and frontline healthcare providers (HCPs); along with concerns about the quality and safety of patient care. The presence of family members and other caregivers as essential partners in care is an explicit expression of the philosophy of patient- and family-centred care (PFCC) in action. The purpose of this study is to increase our understanding of how changes to family presence and visiting policies and practices during the COVID-19 pandemic have impacted patients, family caregivers and frontline healthcare providers (HCPs) in acute care hospitals.

**Methods:**

A total of 38 in-depth semi-structured interviews were conducted with patients, family caregivers and HCPs in Canadian provinces who had experience with visiting policies in acute care settings during the pandemic. COVID patients, and the caregivers of COVID patients, were excluded from this study. A maximum variation sampling strategy was used to guide the selection and recruitment of patients, family caregivers and HCPs, based on our interest in gaining a diversity of perspectives and experiences.

**Results:**

Many patients, family caregivers, and HCPs view family caregiver presence as integral to PFCC, describing the essential roles played by family caregivers prior to the pandemic. There were commonalities across all three groups with respect to their perspectives on the impacts of restrictive visiting policies on patients, family caregivers and HCPs. They fell into four broad integrated categories: (1) emotional and mental health; (2) communication and advocacy; (3) safety and quality of care; and (4) PFCC, trust in the healthcare system, and future decisions regarding accessing needed healthcare. Recommendations for pandemic visiting policies were also identified.

**Conclusions:**

The findings from this study highlighted several impacts of restrictive family caregiver presence or visiting policies implemented during COVID-19 on patients, family caregivers and HCPs in acute healthcare settings across Canada. Participants emphasized that there is no “one-size-fits-all” caregiver presence policy that will address all patient needs. To be consistent with the practice of PFCC, patients and family caregivers are welcomed as part of the healthcare team in ways that work for them, demonstrating that flexibility in family presence and visiting policies is essential.

**Supplementary Information:**

The online version contains supplementary material available at 10.1186/s12913-023-09248-3.

## Introduction

The World Health Organization (WHO) declared COVID-19 a global pandemic in March 2020 [1]. To date (17 December 2022) there have been 4,476,968 reported cases across Canada, with 47% of current cases among young adults aged 20–29 years old [2] and 29.4% of hospitalized patients among individuals aged 80 years and older [2]. The pandemic has entailed numerous challenges for the healthcare system and the care for patients.

The WHO implemented interim guidance for infection prevention and control in healthcare facilities, which included guidance for healthcare facilities to implement policies to limit essential care partners from entering healthcare facilities to protect them from getting infected and reduce their potential to introduce the COVID-19 virus into the health facility [3]. The recommendations put forward acknowledge the need to balance strong measures for infection prevention and control in health facilities against the importance of visits by family members and loved ones for patients’ well-being [3]. The WHO guidance on the implementation of safe visiting policies in healthcare facilities included: restricting movement of visitors within the health facility; encouraging family members to assign a single caregiver or family member who is not at high risk for severe COVID-19 to be with the patient; designating an entrance that visitors who are caregivers or family members can use to access the facility; maintaining a record of all visitors to the facility; and educating and supervising caregivers or family members on proper public health measures such as, hand hygiene, respiratory etiquette, physical distancing, the use of personal protective equipment (PPE), and how to recognize the signs and symptoms of COVID-19.

In accordance with the WHO’s recommendations, the Public Health Agency of Canada (PHAC) published their recommendations on April 30^th^, 2020, prepared by the National Advisory Committee on Infection Prevention and Control, for acute healthcare facilities to develop and implement restricted visitation policies to help prevent the spread of COVID-19 and conserve PPE [4]. Similarly, the PHAC recommendations included limiting visitors to those who are essential, limiting visitor movement within the facility, ensuring a hand hygiene program is in place, and screening every visitor entering the facility [4]. Both the WHO and PHAC recommendations were intended to provide guidance to healthcare facilities to develop their own visitation policies aligned with provincial, territorial, and local legislation and mandates.

Accordingly, throughout the pandemic many Canadian hospitals made significant changes to their ‘open family presence’ and ‘visitor’ policies, particularly in the early days of the pandemic, and instituted restrictive or ‘zero visiting’ policies in acute care facilities, to minimize the spread of the virus. Such policies appeared focused on society’s utilitarian responsibility to mitigate transmission into and within hospitals, and to preserve PPE when concerns existed about access to its supply. Despite decades of advocacy and the supporting evidence, the public discourse on restricting families and caregivers within acute care hospitals throughout the pandemic has overlooked the principles of patient-and-family-centred care approaches. Moreover, the distinction between *visitor*s and *partners in care* has generally not been made; rather the implementation of restrictive visiting policies throughout the pandemic has supported a dominant narrative of families as visitors. Evaluation of the negative consequences of restricted family presence and visitation policies on hospitalized patients, families and providers and potential efforts at mitigating negative effects are important for ongoing pandemic planning and for other events that may be associated with strain on healthcare systems.

### Benefits of Family and Caregiver Presence in Care

For hospitalized patients, family caregivers play a vital role during their stay. There is clear evidence that the presence and engagement of patients in their care, and partnership with family members and family caregivers (‘family’ as designated by patients) improves patient experience, safety, and health outcomes [5–8]. Research has demonstrated that the presence and participation of family members and caregivers—as partners in care—results in cost savings, enhances the patient and family experience of care, improves management of chronic and acute illnesses, enhances continuity of care, and prevents hospital readmissions [9–13]. Research also shows that isolating patients from the people who know them best places them at greater risk for medical error, emotional harm, inconsistencies in care, and costly unnecessary care [14, 15]. For decades children’s hospitals in Canada and across much of the world, have not considered parents and other close family members or caregivers as ‘visitors.’ Rather they are thought of as partners in caring for their child, and part of the healthcare team [16]. For older patients, hospitalization for acute or critical illness is often associated with reduced cognitive function [17]. Involving families and other partners in care, who tend to be much more keenly aware of any change in cognitive function than hospital staff, are a valuable resource during hospitalization. Furthermore, family members and caregivers are crucial in aiding patients who have problems communicating for a variety of reasons, such as people with complex health issues, disabilities, and for those who have experienced past trauma from the healthcare system.

### Study context

This study was conducted in collaboration with Healthcare Excellence Canada and the IMAGINE Citizens Network. Healthcare Excellence Canada is a new organization resulting from the merger of the Canadian Patient Safety Institute and the Canadian Foundation for Healthcare Improvement, an independent, not-for-profit charity, funded by Health Canada. The organization works with diverse partners to shape the future of quality and safety and build a better healthcare system with, and for, everyone in Canada. Imagine Citizens Network is an Alberta-based, independent citizen-led organization that brings together a network of people- and community-oriented partners who amplify the voice of Albertans in healthcare system reform and envision a healthcare culture where people and their families come first.

### Study objectives and research questions

The main objective of this study was to increase our understanding of how changes to family presence and visiting policies and practices in acute care settings during the COVID-19 pandemic that restricted presence among family caregivers or essential care partners in hospital, have impacted or had negative consequences on patients, family caregivers and frontline healthcare providers (HCPs) in Canadian provinces, where initial rapid changes in visiting policies occurred. The following research questions guided the study: (1) what are the impacts of COVID-19 family presence and visiting policies and practices in acute care hospitals, on patients, family caregivers, and frontline HCPs (e.g., physicians, nurses, social workers)?; and (2) what recommendations do patients, family caregivers and HCPs have regarding acute care family presence and visiting policies?

### Conceptual and theoretical framework

Patient-and Family-Centred Care (PFCC) is the overarching conceptual lens used to guide this research. There are four core principles of PFCC: respect and dignity, information, participation and collaboration [18]. PFCC is an approach to the planning, delivery, and evaluation of healthcare that is grounded in a mutually beneficial partnership among patients, families, and healthcare professionals [18]. PFCC embodies an approach that adopts the perspectives of individuals, families and communities, respects and responds to their needs, values and preferences and sees them as essential members of the healthcare team and are key partners in ensuring safe and quality care [18]. In the PFCC approach, healthcare professionals work in partnership with families to facilitate patient-caregiver shared decision-making regarding the patient’s healthcare management [18]. Family is understood as fundamental to the health, wellbeing and recovery of patients according to the PFCC approach, and family presence is supported based on the preferences of the patient. COVID-19 related restrictive family presence and visiting policies in acute care facilities changed how family members, caregivers and healthcare providers (HCPs) navigate PFCC [19]. The socio-ecological model provides the theoretical basis for understanding the relations and the interaction between patients, their familial and community contexts, and the health care system [20]. According to ecological models the patient or individual is in the center; the microsystem includes family, friends, and loved ones; the exosystem includes the healthcare system, while the macrosystem includes laws, policies and social and cultural values of the wider society [20]. The systems in the ecological model are interdependent of each other, thus by changing policies and practices in one system (e.g., healthcare system), the other systems (e.g., patients and families) can be affected.

## Methods

A qualitative descriptive research design was employed for this study [21]. Qualitative descriptive design is grounded in the principles of naturalistic inquiry representing the view that reality exists within various contexts that are dynamic and perceived differently by people; therefore, reality is multiple and subjective [22]. The goal of qualitative descriptive research is to to obtain ‘straight descriptions of a phenomena’ and provide a comprehensive descriptive summary of the experiences and perceptions of a group of people, without abstract rendering of data [22]. In the current research, this translates into understanding how changes to family presence and visiting policies and practices in acute care settings during the COVID-19 pandemic have impacted patients, caregivers, and frontline HCPs in Canadian provinces. This qualitative design is appropriate to facilitate an exploration of patient, family caregiver, and HCP perspectives and experiences with restrictive family presence or visiting policies from their own stories.

### Data collection and participants

Data was collected from key informant interviews who were identified through existing networks of Healthcare Excellence Canada and Imagine Citizens Network. A recruitment poster was distributed through these networks, which included a contact number for interested individuals to participate in an interview. We conducted 38 semi-structured one-hour interviews via phone or videoconference with patients, family caregivers, and HCPs across Canada, between September 2020 and January 2021. In total, 8 patients, 18 family caregivers, and 12 HCPs participated. Interview participants were recruited from Alberta (*n* = 15), British Columbia (*n* = 7), Ontario (*n* = 7), New Brunswick (*n* = 6), and Saskatchewan (*n* = 2) (see Tables 1, 2 and 3 for key informant profiles). COVID patients, and the caregivers of COVID patients, were excluded from this study. Two researchers (GM, KF) conducted the interviews with patients, family caregivers and HCPs. These researchers did not have any prior relationship to the key informants, except for one patient informant who was known by GM. Of the individuals who responded to the invitation, all agreed and consented to participate except for two HCPs who had initially been in contact with a member of the research team but were not able to schedule an interview time. The number of key informant interviews conducted with family caregivers, patients and HCPs was informed by data saturation. For each participant category we began to hear similar ideas among informants, signifying that data saturation was being reached. There was a strong response rate among family caregivers, particularly from Alberta, to participate in an interview and share their story. A diversity of perspectives and experiences were captured across age, sex and gender, and ethnicity [23]. For sex and gender-based considerations, non-gendered language was utilized when consulting and collaborating with informants to ensure inclusivity and accessibility.

**Table 1:** Patient participant demographics (*n* = 8)

**Province**	**Education level**
AB—3 (38%) BC—1 (13%) NB—2 (25%) ON—2 (25%)	College/technical diploma/certificate—1 (13%) University degree—6 (75%)
**Age**	**Patient advisor experience**
25–44—4 (50%)	Yes—6 (75%)
45–64—3 (38%)	No—1 (13%)
**Gender**	**Prior hospital experience**
Male—2 (13%)	A lot—7 (88%)
Female—6 (75%)	Very little—1 (13%)
**Geography**	
Large City—7 (88%)	

^1^1 Patient did not complete the demographic survey

**Table 2:** Family caregiver participant demographics^a^ (*n* = 18)

**Province**	**Geography**
AB—7 (39%) BC—3 (17%) NS—2 (11%) ON—4 (22%) SK—1 (6%)	Small town/village—1 (6%) Suburb—4 (22%) Large City—9 (50%) Rural—1 (6%)
**Gender**	**Education level**
Male—1 (6%) Female—17 (94%)	College/technical diploma/certificate—6 (33%) University degree—10 (56%)
**Age**	
25–44—4 (22%) 45–64—7 (39%) 65 +—3 (17%0	

^a^4 family caregivers did not complete the demographic survey

**Table 3:** HCP Participant demographics (*n* = 12)

**Province**	**Geography**
AB—5 (42%)	Rural—1 (8%)
BC—3 (25%)	Small town/village—2 (17%)
NB—2 (17%)	Large city—5 (42%)
ON -1 (8%)	Suburb—1 (8%)
SK—1 (8%)	Other 1 (8%)
**Age**	**Job Title/Role**
18–24—1 (8%) 25–44—6 (50%) 45–64—3 (25%)	Senior Level Manager—2 (17%) Nurse—5 (42%) Physician—4 (33%) Social Worker—2 (17%)
**Gender**	**Length of practice**
Male—0 Female—12 (100%)	< 5 years—2 (17%) 6–10 years—2 (17%) 10 + years—8 (67%)

^1^2 HCPs did not complete the demographic survey

Invitation letters and consent forms were sent to key informants describing the purpose of the study and expected outcomes of the study. The interview guide included questions about how the restrictive visiting policies put in place during the pandemic impacted the care experience of patients, family caregivers and HCPs (Additional files 2, 3 and 4). Impacts of interest that were explored during the interviews included: patient and caregiver experience, patient safety, quality of care, mental health and emotional health, transitions in care, patient outcomes, and moral distress. In addition, interview participants were asked about their pre-pandemic experiences with essential caregiver roles, their perspectives on current restrictive visiting policies, and their recommendations for future family presence or visiting policies within acute care facilities.

### Data management and analysis

All interviews were audio-recorded and transcribed verbatim with both the signed and verbal informed consent of the informants. Key informant interviews and field notes were analyzed using QSR NVivo 12 software, to facilitate data management and to enhance the systematic organization and examination of the data. Data analysis was performed by members of the research team (SM, GM, KF) and followed Braun and Clark’s (2006) six-phase methodology to understand and identify the themes of patients, family caregivers and HCPs experiences with hospital family presence and visitation policies [24]. Through this qualitative analytical approach, researchers identify the coded elements from the data through line-by-line analysis and then iteratively develop descriptive themes (coding text directly from transcripts) and interpretive themes (grouping similar descriptive codes together), and then present a final holistic thematic framework of the phenomenon under investigation. As the analysis proceeded, the coding frameworks were compared by the members of the research team until consensus was reached, and the themes that were gradually developed were discussed to ensure agreement and enhance rigour. Additionally, cross-group analysis compared the datasets across the three key informant groups to identify cross-cutting themes. Relationships between demographics, elements of past and current healthcare experiences, and a variety of contextual factors and these emergent themes were also explored. The socio-ecological model was most relevant to this investigation given its emphasis on the interrelationships between individual (micro), interpersonal (meso), institutional (exo) and societal (macro) level factors that shape healthcare experiences. The inclusion of participants’ quotations under their relevant thematic headings strengthened the trustworthiness and confirmability of the data analysis. Following the interviews, informants were emailed a demographic survey (Additional file 1) and offered a gift card to acknowledge their time and contribution to the study.

## Results

### Visiting policies in acute care during the pandemic

Policy scans of provincial and territorial guidance for essential care partner presence and visitor policies have been published by Healthcare Excellence Canada [25]. Table 4 summarizes the findings from the policy scan on the restrictive family presence or visitor policies in acute care settings for the Canadian provinces represented by key informants that coincided with the time of data collection for this study. While it was not within the scope of this study to examine the policies themselves, key informants did describe in their interviews how some elements of the policies had impacted them. For instance, allowing only one or two essential care partners to be permitted in hospital or having to connect with family and loves one virtually. Furthermore, some key informant quotes below highlight some elements of the acute care family presence and visiting policies, and how these policies were interpreted by key informants.

**Table 4:** Description of family presence and visiting policies and guidelines in acute care settings only for the provinces where key informants are located. Source: Healthcare Excellence Canada Policy Scan Report (updated October 21^st^, 2021)

Province	Acute Care Setting Policies/Guidelines
British Columbia	• Essential and social visitors are now collectively referred to as **“visitors”** • As of October 26^th^, 2021, all visitors require proof to show they are fully vaccinated, those who are not will be unable to visit • Visitors shall be screened at point of entry for symptoms • Visitors are required to follow infection prevention and control procedures such as masking and practicing good hand hygiene. Physical distancing is not required, but personal space should be respected • Up to **two visitors** may visit at a time, with exceptions to compassionate care, pediatric care where the limit of two may be removed, and in emergency care where it is limited to one
Alberta	• **Designated Support persons and visitors** are highly recommended to be fully immunized • Visitors are expected to complete health screening before entering, and follow infection prevention and control procedures, i.e., continuous masking, practice proper hand hygiene and physically distance • Generally, **one essential care partner** is allowed, with two permitted in certain circumstances such as end of life
Saskatchewan	• Restrictions are separated into four levels: (i) Recovery Phase: A Safe Progression to Open Family Presence; (ii) Level 1 – Pandemic Restrictions, (iii) Level 2 – Family Presence Restrictions; (iiii) Level 3 – End of Life Reasons Only. Facilities with a level 3 status have the most restrictions • Recovery: **Two family or designated support persons (DSPs)** can be present at a time indoors. Up to four DSPs may be present at a time for the following: intensive care, maternal, postpartum, and pediatric units; end of life or palliative care • Outdoor visits have no limit on DSPs or visitors that can be present at a time • Level 1: Every patient or resident can have two DSPs. One person can visit at a time • Level 2: All patients/residents can designate one DSP. More than one can be designated in: end of life, critical or intensive care units, maternal or pediatric units • Level 3 restrictions: End of life reasons only • Vaccinations are not mandatory for DSPs
Ontario	• **1 or 2 essential care partners** are permitted to visit per patient, visitor restrictions are at the discretion of each Ontario hospital • All care partners must be instructed on infection prevention and control protocol and adhere to these protocols • Care partners are to be screened before entry into facilities • Some Ontario health facilities will now require visitors and essential care providers to be fully vaccinated against COVID-19
New Brunswick	• No visitors are allowed at this point of time with exceptions for designated support persons (DSPs) • Effective November 19, 2021, DSPs required to be fully vaccinated to visit an admitted patient or to accompany an outpatient • For Emergency Department or Ambulatory Care visits: only one DSP is allowed for patients requiring physical or emotional support, decision-making or communication assistance. This person must be fully vaccinated • **One** DSP is allowed for the following: patients in paediatrics, palliative care, in emergency or ambulatory services, patients hospitalized for more than 14 days • **Two** DSPs are allowed for patients at end of life or patients receiving medical assistance in dying • DSP’s must follow proper infection prevention and control procedure such as washing hands often, wear their masks constantly, comply with physical distancing, and do screening before entering
Nova Scotia	• Effective October 13, 2021, most visitors and support people coming to Nova Scotia health facilities will be required to show full proof of vaccination or medical exemption letter along with government issued ID. Exceptions will be granted for someone accompanying patients under the age of 19, patients in labour and delivery and a patient requiring a substitute decision-maker • For those with an unvaccinated support person, no additional visitors will be permitted • **Three** designated family/primary support person(s) or family caregivers are welcomed for: palliative care, end of life and patients receiving medical assistance in dying • **Two** family/primary support person(s) or family caregivers are welcomed for: Children under 18, patients in ICU, critically ill patients and patients in labour and birth • **One** family/primary support person or family caregiver is welcomed for: hospital inpatients, patients in emergency departments, prenatal visits, and ambulatory care • Hospital inpatients are asked to identify three support people per week. These three designated support may visit each day, but only one will be permitted to visit at a time • Palliative patients and others nearing end of life may identify a maximum of five designated support people. Three of these five designated support people may visit each day and can visit at the same time where space permit

### Essential caregiver roles

The essential caregiver roles described by key informants were grouped into five main categories: (1) provide emotional support, love, comfort and companionship; (2) advocate for patient’s needs; (3) support two-way patient communication with the healthcare team, including sharing important medical history and information; (4) support healthcare decision-making; and (5) provide physical care, including nutritional support. These roles align with the concept of family caregivers as valuable members of the patient’s healthcare team and illustrate the many ways in which they support the care for and recovery of their loved ones.

Key informants described how restrictive acute care visiting policies put in place during COVID-19 interrupted these essential caregiving roles. Patients described the important roles their family caregivers played prior to the pandemic during hospital visits and stays, with many emphasizing the value of companionship, emotional support, love, and compassion. While family caregivers described similar roles, some had emphasized that communication, advocacy, and provision of physical care were critical roles they provided, particularly for patients with cognitive impairment or communication difficulties, and/or complex physical needs.

### Impacts of restrictive visiting policies

**Fig. 1:**
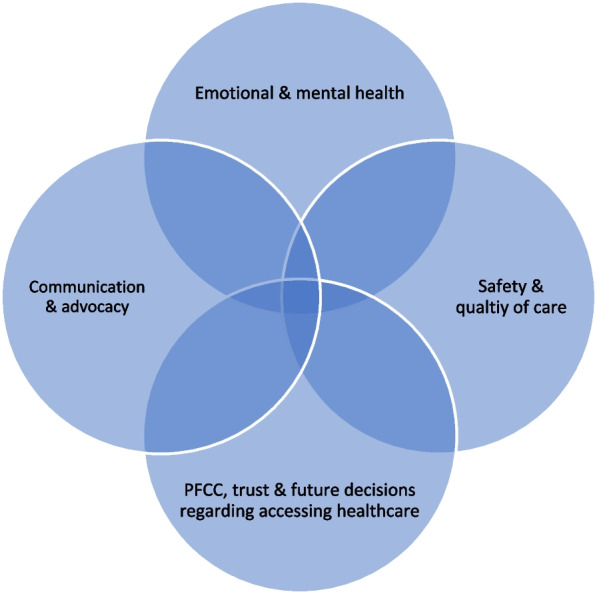
Impacts of restrictive ‘visiting’ policies on patients, caregivers, and healthcare providers

The impact of the strict ‘zero visitor’ policies implemented at the beginning of the pandemic, and ongoing restrictions, has been devastatingly negative for many patients and family caregivers, as well as for HCPs [26]. Although some hospitals across the country have relaxed policies to allow for some family presence at the time of key informant interviews, these policies were continuing to create hardship and distress for many. Most participants used the term visiting or visitor policies to describe these policies. Use of the terms family presence and essential caregivers was limited to those patient and family caregiver informants who were active in the ‘patient advisor’ community, and a few HCPs. For those participants that were aware of the distinction between visitors and essential caregivers, there was recognition that the restrictive visiting policies put in place often did not distinguish between visitors and essential caregivers, meaning there was a negative impact on family presence. There were commonalities across all three groups with respect to their perspectives on the impacts of restrictive visiting policies on patients, family caregivers and HCPs, which fell into four broad integrated categories (Fig. 1): (1) emotional and mental health; (2) communication and advocacy; (3) safety and quality of care; and (4) PFCC, trust in the healthcare system, and future decisions regarding accessing needed healthcare. Within each of the categories key informants described their unique experience and perspective on the policies.

#### Impacts on emotional and mental health

Across all three key informant groups, the theme that emerged most frequently was the impact of these restrictive visiting policies on emotional and mental health. Patients and family caregivers shared their struggles with their emotional and mental health because of the frustrations, stress and anxiety, and isolation due to the restrictive visiting policies. The impact on the emotions and mental health of both caregivers and HCPs, affected patients and the quality and safety of their care.

#### Patients

HCPs, family caregivers and patients all described the impact these policies had on patients’ cognitive, mental and emotional health, describing mental deterioration with respect to anxiety, depression, delirium, and dementia. Separation from loved ones created huge anxiety for patients, and particularly so when the patient had a rare and/or complex medical condition. For patients who had longer hospital stays, feelings of loneliness and isolation were common. A family caregiver described how their loved one fell into a depressive state from being isolated and apart from family:

*“It was extremely hard for him. He went into a very depressive-like shutdown state where he like stopped talking to people. He got really irritable with people like doing the vital checks and stuff. Like, just simple tasks became very, very difficult for him to do.” (08, caregiver)*

One patient shared their experience during a multiple-week hospital stay that included a long stay in ICU. They experienced problems with severe and long-lasting dementia, which they believed could have been lessened by having family members involved in their care. This patient explained,

*“I think for critically ill patients, or patients coming out of surgery...or even patients with dementia and stuff like that, where it’s a confusing time for people...where they, like, don’t know where they are... having that person that’s familiar to them, I don’t think you can replace that… So, I think that’s integral, is that familiar person.” (03, patient)*

An additional burden described by some patients was the impacts that these restrictive hospital visiting policies had both on their family caregivers, and on loved ones and friends that were unable to visit. Patients then, also experienced increased caregiver burden due to these policies, as they worried about the stress it placed on the sole caregivers allowed in hospital. For example, one patient spoke about being worried about her two designated family caregivers becoming exhausted over a multiple week hospital stay, as only two essential care partners were permitted to stay in hospital with her. In past hospital stays it worked well for her to have a team of friends and family who could spend time with her. She described,

*“So, I was only home a few days. And then, I was back [in hospital] for another nine weeks. So, my daughter and [partner] were my two chosen support people. And they rarely missed a day. My daughter would come in the afternoon, and [partner] would come in the early evening…And, um, they were… absolutely exhausted. They were almost as exhausted as I was at the end of the hospital stay…they were the only ones that could come and be with me.” (02, Patient)*

Other patients spoke about being worried about the impact on their children when they were unable to stay with them in hospital, or the impact on other members of a family (e.g., when sibling or grandparents) who could not be present. One patient describes the impacts on her young daughter with special needs from being apart while she was in hospital:

*“…the fallout of that is that she’s had extreme behaviors. And so, we had gone from barely needing any interventions for her, to now we have at least 30 hours a week of professional intervention to handle for not being able to see me just for that little bit of being in hospital.” (05, Patient)*

#### Family caregivers

A predominant theme that emerged was the worry, stress, fear, and loneliness experienced by family caregivers when separated from those they loved. Many described how guilty they felt not being able to be there for their loved one at such a difficult time, and for their inability to offer support. Some family members described sitting in their car and crying after dropping their loved ones off at the emergency department. Others described waiting for hours to hear what had happened to their family member after they were dropped off at the emergency department or post-surgery.

Some family caregivers whose partner or spouse was hospitalized or were the lone parent able to be with their child in hospital, described feeling considerable loneliness and isolation at a time of great stress. This loneliness was often compounded because they were sometimes not able to see anyone else outside their household due to pandemic public health restrictions and felt completely on their own. In some cases, the impacts on family caregivers’ emotional and mental state contributed to deteriorating mental health, including heightened anxiety and depression.

*“I think that was, like, one of the biggest impacts, that you just, kind of, carry the weight… you’re emotionally, and physically exhausted…” (06, caregiver)*

Furthermore, visiting policies that limited access to only one or two essential caregivers could lead to caregiver strain and exhaustion. For example, parents who normally would share this role of supporting their child in hospital now felt entirely responsible for the care of their ill child. Parents described the pressure they put on themselves to notice everything that did not seem quite right with their child, and to remember to ask HCPs all their questions. This included parents of children with complex health and care needs, where care responsibility is usually shared by both parents and sometimes paid caregivers. One family caregiver said,

*“And you’re just exhausted, right? So you’re trying to be a really good listener and communicator between your spouse or your partner, and the healthcare team who’s coming and going” (06, caregiver)*.

#### Healthcare Providers

HCPs spoke about the complex and entangled ways that both COVID-19 and the resulting restrictive family presence and visiting policies affected them and their ability to do their work, which was also observed by patients and family caregivers. These impacts fell into three overlapping categories: role strain, burnout or compassion fatigue, and moral distress, all of which affected their emotional and mental health as well as their ability to provide high quality and safe patient care.

As noted earlier, family caregivers play essential roles for patients during hospitalization. With restrictive family presence in hospitals during the pandemic, HCPs were spending more time with patients and meeting their needs. They also had to adapt to changing visiting policies and procedures during the pandemic and expressed frustration with the added time required to explain visiting policies to families and monitor visiting (e.g., who is the designated caregiver, the times the caregivers are allowed to visit, visitor sign in/out, hand hygiene, use of PPE) as explained by a nurse:

*“We spend certainly, more time communicating policies and restrictions. And then oftentimes finding that we have to defend those policies and restrictions to patients and families… I think has been quite time consuming […] it takes you away off the floor, away from your patients when you’re on the phone with their family members and not just one family member, it’s... You know, you have 25 patients, and sometimes they have multiple children or people that are calling concerned and expressing their own frustrations.” (10, HCP)*

Patients and family caregivers also spoke about their views on how the combination of these restrictive policies and COVID-19 overall affected HCPs. The most dominant theme was the observation about how staff were “run off their feet”, in part because now care delivery was much more difficult. Family caregivers also described experiences where HCPs “worked around” the restrictive visiting policies to meet the needs of patients and their families. Some family caregivers described the impact on healthcare staff when visiting policies evolved to allow some family presence.

*“ I found while being an inpatient [as a parent caregiver], it was very difficult…Like, the staff is so busy that I felt just horrible asking them for just basic things like water or to heat up my meal, or just anything like that. Like, I felt just horrible because they’re so busy… They’re already overwhelmed with all the new rules and regulations due to COVID.” (08, family caregiver)*

The role strain being experienced by HCPs contributed to burn-out and compassion fatigue. Some described taking extended leaves from work because of burn-out, and others contemplated resignation. The following quote from a nurse illustrate this:

*“I went home from some shifts thinking to myself, I feel like I have just worked the heaviest patient load shift I’ve ever had and yet I only had a handful of patients. But it was all of the emotional toil and work on some of those shifts, dealing with all of these dynamics of visitors and family.” (06, HCP)*

HCPs acknowledged that the restrictive visiting policies were not aligned with their usual practice of PFCC, creating considerable moral distress for them and their colleagues. They faced moral dilemmas between what they thought was best practice, best for the patient and what the policies and rules were. As one nurse stated, *“a lot of my colleagues are very morally distressed about having to have families split up and not being able to provide, sort of the family-centered care that we really pride ourselves on” (03, HCP).*

Furthermore, some HCPs described risking their job to make exceptions for bringing in families and caregivers to be with their loved ones, especially in palliative care situations, and echoed by family caregivers. One physician described,

*“An exception I made is a patient who needed palliative sedation and before we put him into coma with the palliative sedation, I snuck up both his sons, daughter and wife to be in the room with him for the afternoon. I said to the nurses, don’t look, don’t watch what I’m doing. If I get in trouble and get disciplined and I’m disciplined and you’re not, I’m at the end of my career, you’re at the beginning. But what would you do ethically? Like it honestly causes a lot of moral distress.” (07, HCP)*

#### Impacts on communication and patient advocacy

Restrictive visiting policies affected communication between patients and family caregivers, other family members, and members of the healthcare team. Family caregivers supporting patients who were unable to effectively advocate for themselves and/or had challenges communicating were particularly worried about the potential impact of these visiting policies on their ability to communicate with members of the healthcare team, and advocate for their loved one. This included children and youth with complex health issues, people who were severely ill, and elderly people. In some cases, the family caregiver also had power of attorney and medical decision-making authority, making this particularly essential, as this patient stated:

*“The power of attorney needs to be present because there’s decisions to be made…So being intubated, you’re unconscious, you’re in an [induced] coma… you really need somebody to speak for you.”* (07, patient)

Patients who had complex health issues and medical histories, and those that had experienced past healthcare trauma, also identified caregiver support for advocacy and communication as being critically important for them and described how it was negatively affected by the visiting policies. One patient who experienced a high-risk pregnancy expressed how the policies impacted communication and advocacy for herself:

*“I did feel like COVID policies, [were] not only making it difficult to communicate… be with and communicate with my family about my health… But it also made me feel like wishes [and]… knowledge I have about my own body and about how I wanted to birth… was not going to be taken into account…” (08, patient)*

Family caregivers also spoke about the difficulties they experienced trying to speak with their loved ones, when they were not allowed to be at the bedside. Connecting with members of the healthcare team, and receiving information around diagnosis and patient status, was described by many key informants as challenging. A daughter who was caring for her father, in partnership with her mother, stated:

*“my mother, sometimes she would have people [referring to HCP], say oh, he’s doing the same as yesterday, and hang up on her. My mom would say, well can you please tell him that his wife has called and said she loves him...” (05, family caregiver)*

HCPs also spoke of this when they described the role strain associated with ensuring patients and family caregivers were connected and updated with their loved one’s status. A nurse manager explains,

*“It felt very unsafe for our desk because they [nurses] would be trying to manage a busy acute unit, and literally have multiple phones calls every five minutes, of people trying to connect to their family members. And there were many days where I went to the desk and sat there with the clinician and the unit clerk, and we just answered the phone for an hour, just to let them [nurses] catch up with their work…. We could have had a full-time staff member just running a phone, back and forth.” (04, HCP)*

One way that HCPs and families tried to overcome communication challenges was using technology mediated devices, such as a tablet. Several patients explained that using a phone, tablet or computer to connect remotely with loved ones was not helpful or even possible. This was described by patients who owned a cellphone or tablet but were not accustomed to using video or text messaging and/or had a cognitive or physical disability that prevented them from communicating with others in this way. Connecting patients with their caregivers through technology, then, often required assistance from HCPs, something which HCPs did not always have the time or capacity to support.

HCPs also described the challenges some patients had connecting remotely with family and loved ones. One social worker stated,

*“People were lonely and, I mean, we put in place the Facetime visits and window visits in the facility. So, we had iPads and they would have calls, family would call occasionally. But maybe only like 10% of patients there were able to interact using technology. Many of our patients have dementia… they’re also much older and they’re not used to this technology”* (01, HCP)

One family caregiver describes the difficulties she encountered when trying to connect with her husband who has early onset Alzheimer’s while he was in hospital.

*“So, I finally got to somebody, and I explained, you know, what my concern was. I said I haven’t heard from him. I can’t hear from him. He doesn’t have a cell phone. He can’t use a cell phone[…]Uh, so they finally took a phone to his bedside and that was more confusing, I think, than anything because he didn’t really... He could hear me on the phone, but he didn’t understand... You know, he wanted me to come to him.” (14, family caregiver)*

HCPs also commented on how patient advocacy and communication was affected by the restrictive family presence or visiting policies, and how this in turn could affect the patient’s safety. One physician described,

*“It’s never good to be in a system of telephone, where one person gets told something and then they go and tell one other person and so on. That’s why, when we normally have family meetings, we normally have lots of family representation there. Essentially, whoever the family wants to invite. I mean, there’s a lot of safety in a family, a group of people, hearing the same message that they can then, sort of, reinforce to each other and clarify with each other. I think that has a lot of value …”* (03, HCP)

The challenges described about communication and patient advocacy greatly contributed both to the emotional and mental health impacts on patients, family caregivers, and HCPs. The inability of family caregivers to communicate with the patient, even to simply tell them they are loved, can be very distressing for family.

#### Impacts on safety and quality of care

Patients and family caregivers shared examples about how a lack of family presence affected safety and quality of care for patients. The concerns about safety primarily related to falls, positioning, and medication errors. The lack of family caregivers’ presence to support communication and patient advocacy was seen as a major contributing factor. For example, sometimes patients were unable to communicate that they were in pain, and problems with communication at discharge could create potential unsafe situations. One caregiver described their family member arriving home from hospital with a drug prescription written for another patient. Another caregiver described how worried they were when having to drop their ill and frail spouse at the entrance to the hospital, knowing they had to walk a distance to get to the clinic they were attending. She was concerned her spouse might fall.

Many patient and family caregiver descriptions about a lack of quality of care related to the personal and physical care a loved one received during their hospital stay. This included being washed or bathed, having their teeth brushed, and getting adequate nutrition and hydration. Additionally, other key informants attributed poor quality of care to being unable to participate in outpatient appointments, such as cancer treatments or clinical consultations. This meant that family caregivers were not present to be that second set of ears and to ask important questions, which can result in both unnecessary and delayed testing or care. Some family caregivers expressed serious concerns about quality of care when patients were unable to communicate and/or advocate for themselves. As described earlier, one patient with a rare condition had to call their spouse to come and pick them up after their surgery, as they were struggling to make themselves heard without family caregiver help to advocate for their needs, creating a potentially life-threatening situation for the patient. HCPs also spoke about patients not receiving quality care or the standard of care, and shared examples of how the visiting restrictions put in place jeopardized patient safety as described by one nurse:

*“I think patients are safest when their families are here. I think the hospital setting is absolutely overwhelming. It becomes such an unsafe time, in terms of information transfer, in terms of getting questions answered, in terms of advocating and bringing up all of those nuances of who the patient is and what’s going on. Without family there to be that advocate and stand beside them. I think it’s always a dangerous time when they’re not here.” (06, HCP)*

Safety concerns were also shared by HCPs, as noted by one palliative care physician who described that some patients are not wanting to stay in hospital because of the visiting policies during the pandemic, which led to several injuries and other complications in the home.

*“In hospital we sedate patients who experience delirium and treat depression. At home, because these patients aren’t coming into hospital, we have somebody with delirium that really needs nursing expertise to manage medications for the delirium. I’m giving medications to patients in the home with families to administer and I have had three patients who have then had very nasty fractures because they’ve fallen and it’s using medications that families aren’t equipped to manage or deal with. And yet they want to be with their families. They want to be with their loved ones.” (07, HCP)*

HCPs often referenced role strain and lack of time as reasons why the quality of care for patients has been compromised during the pandemic. One nurse described how staff were trying to carry out their care duties in a single visit with the patient to save time, and that this impacted quality of care for patients:

*“We are guilty for it, where it’s like, if you’re going in with medications or to do a dressing change or vitals, to interact with them, or to bring them their meal tray, like, one-stop shopping, just to limit that donning and doffing, and then it’s like, okay, is there anything else that you need while I'm in here? And that they do have fewer contacts with their health-care provider. You’d be doing your rounds and checking in on them, but […] you know that care is impacted by the visiting policies that are in place.” (10, HCP)*

#### PFCC, trust in the healthcare system, and future decisions regarding accessing needed healthcare  

As described previously, many HCPs, and some patients and family caregivers recognized that the visiting restrictions put in place during COVID-19 created tension with the concept of PFCC. This tension contributed to the frustrations with the visiting policies described earlier, and the strong advocacy efforts mounted by some HCPs, family caregivers and patients to have family members present. It was one source of moral distress experienced by HCPs, in that they felt unable to provide PFCC in the way they previously had and wanted to.

Patients and family caregivers familiar with the concept and language of PFCC commented on how visiting restrictions that affected their ability to have family and loved ones critical to their recovery and wellbeing present while they were in hospital, was the antithesis of PFCC. These restrictive policies precluded meeting patients and families where they were at and determining with them what would work best for them during a hospital stay or visit during the pandemic. For some, this contributed to further erosion of their trust in the healthcare system, and particularly for those who have experienced some healthcare trauma in the past. An impact of this erosion in trust was fear about needing to access hospital care during the pandemic if there were family presence restrictions in place.

“*My husband was willing to abide by every masking rule, PPE rule, whatever they said [referring to allowing her husband to be present at the hospital], but none of those things were asked. And in traditional patient-and-family-centered care, you would have asked that…” (patient, 05)*

Some patients and family caregivers described how restrictive family presence policies had affected, or would affect, their decisions about accessing hospital services. For example, one family member described the difficult decision they had to make with respect to taking her sibling with complex care needs to hospital after she had a fall, as they knew how incredibly difficult this would be both for that person and the family. They had delayed, hoping that they would not have to take them, but eventually there was no alternative.

*“So when we decided [they were] going to…have to go to the hospital, my mom really struggled…We’ve had some poor experiences in the past at the hospital… and we just knew that perhaps only one of us could go. And my [sibling’s] physical needs are also very high…And so often they require two people to just visit because [they don’t] understand. And so doing something like an X-ray or blood work is very traumatic for [them] and very challenging…And it often requires two of us.” (11, family caregiver)*

*“It was a trade-off. And if she had gone to hospital, she would have probably been given antibiotics by IV…and she would have had a chest X-ray, and, um, she, she might have made it through…But the trade-off wasn't worth it...to have my mother die alone in a hospital...” (18, caregiver)*

### Policy Implications and Recommendations

Because of the impacts of these policies experienced by patients, family caregivers and HCPs, inviting their perspective on what family presence policies could look like in a pandemic, was important. Key informants provided both specific policy suggestions, and what they felt were key underlying principles of these policies. These recommendations are summarized in Table 5.

**Table 5:** Policy recommendations

Underlying principle	Specific recommendations^a^	Quotes
Balance the risk of COVID contagion with potential harms of restrictive policies	-----Take an evidence-informed approach to balancing the risks of infection, rather than taking a reactive approach to policy development driven by fear	*“[these restrictive policies have been] very reactive and fear-driven. And that might be okay for the first week [of the pandemic], but we can’t keep going on like this…And I kept thinking, okay, what’s the risk, versus what’s the harm that you’re causing?”*
Return back to policies that support patient and family-centred, compassionate care	-----Allow both parents and/or others (e.g., grandparents) to support children in hospital -----The presence of a support person or caregiver, as defined by the patient and their family, is essential not simply ‘nice to have’ -----Re-enforce PFCC at the systems level by creating metrics to measure and evaluate PFCC; include family and caregivers as essential and part of the multidisciplinary patient care team (HCP) -----Change the language from visitor to essential caregiver (HCP)	*“I think before COVID hit we were at a place where… And I think it, it shows in some of my own experiences in acute care, where family was respected and welcomed. And I think that brought us together. You know the saying together we’re better? … we need to recognize that and not go backwards in time.”*
Ensure flexibility regarding how family presence is defined and supported	-----Remove blanket policies, allowing for some local hospital autonomy including for extraordinary cases (HCP) -----Incorporate flexibility into visitor policies by taking into consideration contextual factors with families (e.g., if there are more than 1 family caregivers) as well as the patients’ health status (e.g., palliative, dementia, mental health concerns). (HCP)	*“More than anything, I think the underlying policies need to listen to the patient […] and ask the patient, who are those essential people for you.” (05, Patient)* *“It’s hard to have it so cut and dry, uh, where everyone follows the same rules. I think that, you know, we can have some guidelines for sure, but I think there needs to be a humane, um, side to it.”*
Support family members to be physically present, when desired	-----Put in place practices to support safe family presence (e.g., good screening criteria, rapid on-site testing, access to appropriate PPE) -----Have manpower to educate family caregivers around infection control and policies, and monitor the safe practice of these policies (HCP)	*“I think, if they could… Now that they're looking at staffing, um, to ensure that there's someone always monitoring the visits to make sure the masks are on, that they clean their hands, that they're not eating and sharing food with their loved one”*
Foster consistent understanding and implementation of policies	-----Ensure policies are transparent and well communicated, especially as the policies are changing throughout the pandemic -----Work with HCPs to understand how best to communicate new policies and procedures (HCP) -----Have a designated coordinator to support monitoring of visiting procedures	*“… speaking to frontline staff who, um, you know, before they, they finalize the policy, what’s the impact of this policy? What’s it going to really be in real terms on the floor? GA Yeah. Yeah. LA Because, you know, policymakers can be pretty theoretical in their, their thinking, and not, necessarily, be in touch with front line.”*

^a^Specific recommendations that came from HCPs are noted in brackets. All other specific suggestions came from patients and family caregivers

## Discussion

In Canada, over the recent years, there has been a significant increase in accommodating visiting policies, from 32% of hospitals having such policies in 2015 to 73% in early 2020 [26]. Following the onset of the pandemic, these same hospitals suspended or significantly limited family presence access [8, 26]. Considering evidence that demonstrates benefits of family/essential caregiver presence to patient care, this study sought out to examine the impacts of acute care restrictive family presence and visiting policies in Canada during COVID-19. The findings from this study highlighted the perspectives and experiences of patients, family caregivers, and HCPs on the impacts of restrictive acute care family presence or visiting policies on their emotional and mental health, communication and patient advocacy, safety and quality of care, and trust in the healthcare system. There were commonalities across all three groups with respect to their perspectives and experiences on these impacts. Barriers at the societal (e.g., society’s utilitarian responsibility to mitigate COVID-19 transmission), policy/institutional (e.g., restrictive acute care visiting policies to contain the spread of the virus), interpersonal (e.g., inadequate formal and informal support for patients in hospital due to limited family caregiver presence and strain on the healthcare system), and and intrapersonal levels (e.g., moral distress among HCPs on compromising patient safety and quality of care) contributed to participants having negative experiences with restrictive visiting policies in hospital during the COVID-19 pandemic.

Our findings on the impacts of restrictive visiting policies during COVID-19 are consistent with research reported by other scholars. Krewulak and colleagues (2021) conducted a similar qualitative study examining the consequences of visiting restrictions during the pandemic and described how the restrictions had a negative impact on the ability of patients and families to have their psychosocial and information needs fulfilled, quality of communication between family caregivers and HCPs, and changing roles and responsibilities with respect to communication resulting in patients communicating information about their health state or condition to family members, potentially leading to misinformation [27].

Key informants described the essential role of care partners in hospital. The desire and need for patients to have family caregivers support them during their hospital stay is influenced by a range of factors. For instance, patients with complex and/or rare medical conditions, and those who have experienced some healthcare trauma, often have a greater need for family support. The reason for hospitalization, the length of hospital stay, and the severity of the patient’s condition were described by patients and family caregiver informants as contributing factors. Family and caregiver desires to be present in hospital also varied considerably. Parents of children, family caregivers of people living with disabilities and/or cognitive challenges, and family caregivers of people with complex and/or rare medical conditions often have a greater need to be present during hospital appointments and stays. In the pediatric literature, family presence has been found to improve hospital safety through increased surveillance and detection of potential medical errors [28]. Family members become essential in communication surrounding escalation of care, particularly in the setting of incapacity [29].

Separation from loved ones or family resulted in feelings of withdrawal, depression, and increasing confusion for some but not all patients who were interviewed. Competent adults who have some hospital experience; feel completely able to communicate with their healthcare team and advocate for themselves; were able to communicate with their family or friends via virtual modalities; and who were in hospital for a short period of time, or are accessing an outpatient service, often felt they did not require any family caregiver presence during their hospital stay. Many of these patients are often more concerned about protecting their loved ones from the COVID-19 virus, and consequently encouraged them to stay away. Should something change, such as if they were to become seriously ill or felt for any reason that they could no longer advocate for themselves, then their needs would change. So not only is the need for family presence different for different people, what this looks like for a particular patient and family can evolve over time. Moreover, while telephone and video communication has been reported to be an effective alternative way to connect with families and loved ones when visiting is restricted, this may be limited by barriers such as knowledge of technology especially with older individuals and access to devices that enable video conferencing. There is ample evidence demonstrating barriers to accessing digital technologies among underserved and vulnerable populations [30, 31].

Patient populations at high risk of harm from the absence of family/essential caregivers must be identified such as, patients with cognitive impairment, at risk of delirium, patients with communication barriers, critically ill patients and at the end-of-life, and patients with unique sociocultural needs. More research is needed surrounding the impacts of restrictive family presence and visiting policies in hospital across different racialized population groups. Racial disparities and structural racism have been documented across the provision of healthcare in Canada [32–34]. Pandemics also amplify underlying pre-existing health disparities [35, 36]. Patient advocacy has been one mechanism to mitigate the potential for structural racism in healthcare for more vulnerable patients.

Our findings also showed that some patients and family caregivers made the difficult decision to not visit a hospital because of restrictive family presence policies. This suggests another layer of challenges with patient delaying access to healthcare, and the potential impacts this may pose on both the patient and families and the healthcare system.

The hardships experienced by patients and family caregivers because of restricted policies were also experienced by HCPs. The COVID-19 pandemic has placed a heavy toll on HCPs, who grappled with fatigue and burn-out while adhering to visiting policy guidelines, unfamiliar virtual modalities (to connect competent patients with their loved ones), and in acquiring necessary information when family caregivers have been restricted entry into healthcare facilities. HCPs also described feelings of anxiety because they could not provide the best care in a way that was PFCC. Some described risking their job to make exceptions for patients and families. They described many situations where they believed that the safety and quality of patient care was compromised by the lack of family presence. Silvera et al. (2021) also found that hospital performance was negatively impacted from COVID-19 and in particular hospitals with a ‘no visitors policy’ saw the most pronounced impacts which included patient ratings of medical staff responsiveness as well as sepsis and fall rates [37].

Despite a lack of evidence demonstrating that family caregivers or essential care partners played a role in the transmission of COVID-19 in hospitals [38–41], and the growing evidence of the negative impacts of restrictive visiting policies, hospitals across Canada have been slow to adopt more accommodating visiting policies during the pandemic. Interview participants highlighted the importance of balancing risk of COVID-19 contagion with potential harms of restrictive family presence policies and the resultant increase in workload to HCPs in their absence, something that can be achieved by ensuring flexibility regarding how family presence is defined and supported. Early restrictive visiting policies only looked at the harms associated with COVID-19 infection/transmission and did not take a balanced approach to consider the significant range of harms to all involved as clearly articulated in the study findings. The range of experiences and contexts described by family caregivers, patients, and HCPs led many to the realization that there is no “one-size-fits-all” family presence policy that will address all patient needs. It should not be presumed that there is one person, or even two people, who are going to be able to play that essential partner in care role. In the context of restricting presence to essential caregivers only, some described how important it was that determination of who is essential to a particular patient—and when their physical presence is essential—cannot be determined unilaterally by a healthcare organization or by healthcare staff. Rather, in an organization that practices PFCC, this needs to be determined in collaboration with the patient and their family.

### Strengths and limitations

There are several strengths of this study, including the diversity of perspectives and experiences captured from patients, family caregivers and HCPs across different Canadian provinces and hospital settings. The findings also add valuable information to existing published research on the impacts of restrictive visiting policies in acute care settings during the pandemic [27, 42, 43]. Despite these strengths, there are also limitations to this study. Although data saturation was reached, most of the included participants were family caregivers (*n* = 18) and from the province of Alberta (*n* = 15). There were only two participants from the province of Saskatchewan (one family caregiver and one HCP) which may not be generalizable to other family caregivers and HCPs in that province. Furthermore, only six Canadian provinces were captured in this study and the experiences and perspectives of patients, family caregivers and HCPs may not be representative of the entire country. The study population across all three key informant categories was predominately white Caucasian and European, and the perspectives and experiences across different cultural groups were missing. Also, the study findings are limited to settings like Canada where there was 24-h visitation before the COVID-19 pandemic. Lastly, the focus of our study was to examine the impacts of restrictive family presence and visiting policies in Canadian hospitals, thus the findings predominately illustrate negative experiences of key informants with these policies. Nonetheless, the findings are commensurate with strong evidence demonstrating the importance of family caregiver presence in hospital and that COVID-19 related restrictive family presence and visiting policies are associated with potential harm for family members, caregivers and HCPs [26, 43–46].

## Conclusion

The COVID-19 pandemic has necessitated a temporary shift in how healthcare organizations support family presence and visitors. The adoption and implementation of family and caregiver presence policies is rooted in an underlying philosophy of PFCC. While most healthcare settings have traditionally welcomed family members as partners in care with open access to the facility, COVID-19 has prompted risk mitigation strategies that have challenged usual policies around family presence. Although some healthcare facilities across Canada have begun to re-integrate elements of family presence into their policies during this COVID-19 pandemic, ongoing restrictions continue to create great hardship for many. The emergence and ongoing threat of the COVID-19 pandemic has challenged the ability of many healthcare facilities to balance their philosophies and practices of PFCC with safety and infection prevention and control considerations during this time of crisis. The findings from this study highlighted several impacts of restrictive family presence or visiting policies on patients, family caregivers and HCPs in healthcare settings across Canada during COVID-19. These impacts have been devastatingly negative for many patients and family caregivers, as well as for HCPs. Many family caregivers and patients noted there is no “one-size-fits-all” family presence policy that will address all patient needs. To be consistent with the practice of PFCC, patients and family caregivers are welcomed as part of the healthcare team in ways that work for them, meaning that flexibility in family presence and visiting policies is essential. As acute care facilities begin to reintegrate family presence into their facilities, it is imperative that this work be done in partnership with those who have lived experience – patients, family caregivers, and HCPs. Given the role strain and pressures placed on HCPs, healthcare facilities can have in place designated healthcare staff or team to facilitate communication with family caregivers on infection prevention and act as a liaison to support families and patients admitted to hospital. Moreover, further research is needed surrounding the impacts of restrictive family presence and visiting policies across different racialized population groups.

## Supplementary Information

**Additional file 1.** Participant Demographic Information

**Additional file 2.** Interview guide: Patients.

**Additional file 3.** Interview guide: Healthcare providers.

**Additional file 4.** Interview guide: Families/Caregivers.

## Data Availability

The datasets used and/or analyzed during the current study are available from the corresponding author on reasonable request.
