# Supplementary material for: Family caregivers as essential partners in care: examining the impacts of restrictive acute care visiting policies during the COVID-19 pandemic in Canada
Source: BMC Health Serv Res. 2023 Mar 31;23:320. doi: 10.1186/s12913-023-09248-3 (PMC10066017; doi:10.1186/s12913-023-09248-3)
Supplement: Supplementary file 1 — Additional file 1. Participant Demographic Information [file 12913_2023_9248_MOESM1_ESM.zip › Demographics_family_caregiver_Visitation policies TEMPLATE.docx]

**Family Caregivers as Essential Partners in Care: Examining the Impacts of Restrictive Acute Care Visiting Policies During the COVID-19 Pandemic in Canada**

**BACKGROUND & DEMOGRAPHICS SURVEY**

Date (day/month/year): ___________________

*Personal Information*

1. What province do you live in? _______________________
2. How do you describe where you live?

- Remote
- Rural
- Small town or village
- Suburb near large city
- Large City
- Other (please specify) ____________________________

1. What is your age?

- 18 to 24 years
- 25 to 44 years
- 45 to 64 years
- 65 years and over
- Prefer not to answer

1. With what gender do you most closely identify?

- Male
- Female
- Non-binary
- Prefer not to answer
- Other _______________________

1. People living in Canada come from many different cultural and racial backgrounds.

What cultural and/or racial background do you identify with?

_____________________________________________

1. What is your primary language?

______________________________________________

1. What is your highest level of education?

- Some high school or less
- High school diploma
- Some post-secondary without diploma or degree
- College or technical diploma or certificate
- University degree
- Prefer not to answer
- Other (please specify): __________________________
