# Supplementary material for: Family caregivers as essential partners in care: examining the impacts of restrictive acute care visiting policies during the COVID-19 pandemic in Canada
Source: BMC Health Serv Res. 2023 Mar 31;23:320. doi: 10.1186/s12913-023-09248-3 (PMC10066017; doi:10.1186/s12913-023-09248-3)
Supplement: Supplementary file 2 — Additional file 2. Interview guide: Patients. [file 12913_2023_9248_MOESM2_ESM.docx]

**Family Caregivers as Essential Partners in Care: Examining the Impacts of Restrictive Acute Care Visiting Policies During the COVID-19 Pandemic in Canada**

**Interview guide: Patients**

**Introduction**

- The purpose of this qualitative research project is to increase our understanding of the impacts of COVID- 19 visiting policies and practices, put in place in acute care hospitals, both on patients and their families/caregivers [including their impact on family presence] and on frontline healthcare providers.
- Go over the consent form: any questions?
- Ask permission to record: obtain verbal consent.

**Interview questions**

1. Could you tell me a little bit about yourself, and your experience with our healthcare system and hospital before COVID?
2. I understand you’ve had some hospital experience (as a patient) during COVID-19. Could you please tell me about that experience? When did this happen, why and where?

Probe around:

- Was this something you were doing routinely pre-COVID (e.g., ongoing TX such as dialysis, Ca chemo or radiation therapy), or something that happened/started during COVID?
- If you were an inpatient, how long were you in-hospital?
- If you visited the ED, how long was your hospital visit?
- If you were receiving regular outpatient treatment, how often did you need to go to hospital and over what period of time?

1. What ‘visiting’ restrictions (due to COVID) were in place at the time you were interacting with/in the hospital?

Probe around:

- Difference from ‘pre-COVID’ hospital policies

1. How did these restrictions affect your hospital experience? What impact did they have?

Probe around:

- Physical comfort
- Emotional state (e.g., worry, fear, loneliness)
- Your ability to advocate for yourself/communication with doctors, nurses and other healthcare professionals caring for you
- Safety
  - Feeling safe without family members/caregivers in-hospital support?
  - Feeling safe from COVID [if in shared room with other patients and their family members?]
- Confidence in the care plan and quality of care you’re receiving

1. Realizing that we will all be living with COVID for some time, what kinds of hospital-visiting and family presence policies would you like to see put in place?
2. What difference would having these kinds of policies in place make to you? Your family?

Probe around:

- Help you have a stronger voice/be an advocate for yourself
- Help you feel safer
- Help you feel more comfortable
- Help you feel less lonely
- Help you feel less scared
- Help you feel loved
- Help you feel more confident in the care being received
- Help you feel more confident with the discharge planning, and knowing what to do when you get back home

1. Is there anything else you would like add?

**Thank-you!**
