# Supplementary material for: Family caregivers as essential partners in care: examining the impacts of restrictive acute care visiting policies during the COVID-19 pandemic in Canada
Source: BMC Health Serv Res. 2023 Mar 31;23:320. doi: 10.1186/s12913-023-09248-3 (PMC10066017; doi:10.1186/s12913-023-09248-3)
Supplement: Supplementary file 3 — Additional file 3. Interview guide: Healthcare providers. [file 12913_2023_9248_MOESM3_ESM.docx]

**Family Caregivers as Essential Partners in Care: Examining the Impacts of Restrictive Acute Care Visiting Policies During the COVID-19 Pandemic in Canada**

**Interview guide: Healthcare providers**

**Introduction**

- The purpose of this qualitative research project is to increase our understanding of the impacts of COVID- 19 visiting policies and practices, put in place in acute care hospitals, both on patients and their families/caregivers and on frontline healthcare providers.
- Go over the consent form: any questions?
- Ask permission to record: obtain verbal consent.

**Interview questions**

1. Could you tell me a little bit about yourself, and your role at the hospital?

Probe around:

- How long have you worked as a nurse/doctor/other?
- What ward/unit(s) do you work in currently? For how long?

1. What kinds of family presence and visiting policies were in place at your hospital before COVID-19? Approximately how long had these policies been in place?
2. What kinds of policies were put in place at the start of COVID-19? How have they changed over the course of the pandemic (if they have)?
3. How do you think the family presence and visiting restrictions put in place at the start of the pandemic affect the patients that you care for? Could you give me some examples?

Probe around:

- Physical comfort?
- Emotional state (e.g., worry, fear, loneliness)?
- Mental health?
- Communication with doctors, nurses and other healthcare professionals caring for you?
- Safety?
- Quality of care?
- Other?

1. How did these restrictions affect you?

Probe around:

- Your ability to provide good care?
- Emotional state?
- Mental health (e.g., anxiety, depression)?
- Sleep?
- Other?

1. Did you experience any moral distress? *[When policies or procedures are put in place that prevent a healthcare professional from doing what they think is right, this presents a moral dilemma. Moral distress is the emotional state that arises from a situation where you feel that the ethically correct action to take is different from what you are told to do/tasked with doing.^[[1]](#footnote-1)^]* Please describe.
2. Realizing that we will all be living with COVID for some time, what kinds of hospital-visiting and family presence policies would you like to see put in place?
3. What difference would having these kinds of policies in place make to you, and your ability to do your job?
4. Is there anything else you would like say?

**Thank-you!**

1. Retrieved Aug 2020 from: <https://engage.healthynursehealthynation.org/blogs/8/531#:~:text=Moral%20distress%20is%20the%20emotional,that%20presents%20a%20moral%20dilemma.> [↑](#footnote-ref-1)
