# Supplementary material for: Family caregivers as essential partners in care: examining the impacts of restrictive acute care visiting policies during the COVID-19 pandemic in Canada
Source: BMC Health Serv Res. 2023 Mar 31;23:320. doi: 10.1186/s12913-023-09248-3 (PMC10066017; doi:10.1186/s12913-023-09248-3)
Supplement: Supplementary file 4 — Additional file 4. Interview guide: Families/Caregivers. [file 12913_2023_9248_MOESM4_ESM.docx]

**Family Caregivers as Essential Partners in Care: Examining the Impacts of Restrictive Acute Care Visiting Policies During the COVID-19 Pandemic in Canada**

**Interview guide: Families/Caregivers**

**Introduction**

- The purpose of this qualitative research project is to increase our understanding of the impacts of COVID- 19 visiting policies and practices, put in place in acute care hospitals, both on patients and their families/caregivers [including their impact on family presence] and on frontline healthcare providers.
- Go over the consent form: any questions?
- Ask permission to record: obtain verbal consent.

**Interview questions**

1. Could you tell me a little bit about yourself and your experience with our healthcare system and hospital before COVID-19?
2. I understand your family have had some hospital experience during COVID-19. Could you please tell me about that experience? When did this happen, why and where?

Probe around:

- Was this something your family member/you was doing routinely pre-COVID (e.g., ongoing TX such as dialysis, chemo or radiation therapy), or something that happened/started during COVID?
- If your family member was an inpatient, how long were they in-hospital?
- If your family member visited the ED, how long was their hospital visit?
- If your family were receiving regular outpatient treatment, how often did they need to go to hospital and over what period of time?
- Other?

1. What ‘visiting’ restrictions (due to COVID) were in place at the time your family member was interacting with/in the hospital?

Probe around:

- How were these different from ‘pre-COVID’ hospital policies - if known? [i.e., After Covid hit, were there changes to the ‘visiting’ policies at the institution your family member was receiving care? What were these changes? Were these different from the ‘pre-covid’ policies?]

1. How did these restrictions affect your family member’s hospital experience? What were the impacts?

Probe around:

- Physical comfort?
- Emotional state (e.g., worry, fear, loneliness)?
- Mental health (e.g., anxiety or stress)?
- Actual things that happened that made you concerned about their care
- Communication/miscommunication with doctors, nurses and other healthcare professionals caring for you?
- Confusion about care plans/meds?
- Safety
  - Feeling your family is safe without family members/caregivers in-hospital support (e.g., unintended harm such as falls, medication errors, etc.)?
  - Feeling your family members is safe from COVID [if in shared room with other patients and their family members?]
- Quality of care?
- Other?

1. How did these restrictions affect you? Other members of your family?

Probe around:

- Emotional state (e.g., worry, fear, loneliness)?
- Mental health?
- Sleep?
- Ability to care for your family member at home, after discharge?
- Other?

1. Realizing that we will all be living with COVID for some time, what kinds of hospital-visiting and family presence policies would you like to see put in place?
2. What difference would having these kinds of policies in place make for your family member? For yourself?

Probe around:

- The kinds of issues raised by the interview participant in response to questions numbers 4 and 5 above

1. Is there anything else you would like to add?

**Thank-you!**
